# A bibliometric analysis of systematic reviews and meta-analyses in ophthalmology

**DOI:** 10.3389/fmed.2023.1135592

**Published:** 2023-03-02

**Authors:** Yihang Fu, Yuxiang Mao, Shuangyan Jiang, Sheng Luo, Xiaoyun Chen, Wei Xiao

**Affiliations:** ^1^State Key Laboratory of Ophthalmology, Zhongshan Ophthalmic Center, Sun Yat-sen University, Guangzhou, China; ^2^Guangdong Provincial Key Laboratory of Ophthalmology and Visual Science, Guangzhou, China

**Keywords:** bibliometric analysis, systematic reviews and meta-analyses, ophthalmology, publication productivity, research trend

## Abstract

**Background:**

Bibliometric analysis is a quantitative method which applies mathematical and statistical tools to evaluate the inter-relationships and impacts of publications, authors, institutions and countries in a specific research area. Systematic reviews and meta-analyses (SRMAs) are summaries of the best available evidence to address a specific research question *via* comprehensively literature search, in-depth analysis and synthesis of results. To date, there have been several studies summarizing the publication trends of SRMAs in research specialties, however, none has conducted specifically in ophthalmology. The purpose of this study is to establish the scientometric landscape of SRMAs published in the field of ophthalmology over time.

**Methods:**

We retrieved relevant ophthalmological SRMAs and the corresponding bibliometric parameters during 2000 to 2020 from Web of Science Core Collection. Bibliometric analysis was performed using bibliometrix package. Pre-registration and guideline compliance of each article was independently assessed by two investigators.

**Results:**

A total of 2,660 SRMAs were included, and the average annual growth rate was 21.26%. China and the United States were the most productive countries, while Singapore was the country with the highest average citations per document. Wong TY was not only the most productive, but also the most frequently cited author. The most productive affiliation was National University of Singapore (*n* = 236). Systematic reviews and meta-analyses output in most subspecialties had steadily increased with retina/vitreous (*n* = 986), glaucoma (*n* = 411) and cornea/external diseases (*n* = 303) constantly as the most dominant fields. Rates of pre-registration and guideline compliance had dramatically increased over time, with 20.0 and 63.5% of article being pre-registered and reported guideline in 2020, respectively. However, SRMAs published on ophthalmology journals tended to be less frequently pre-registered and guideline complied than those on non-ophthalmology journals (both *p* < 0.001).

**Conclusion:**

The annual output of SRMAs has been rapidly increasing over the past two decades. China and the United States were the most productive countries, whereas Singapore has the most prolific and influential scholar and institution. Raising awareness and implementation of SRMAs pre-registration and guideline compliance is still necessary to ensure quality, especially for ophthalmology journals.

## 1. Introduction

Through comprehensive literature search, critical assessment, and synthesis of all related and trustworthy studies on a specific subject, systematic reviews are more rigorous than traditional narrative reviews, and are regarded as the best summaries of existing evidence (1–3). More importantly, they are capable of updating the knowledge in certain field as well as proposing future research directions (3). Featured by a replicable and methodical presentation and methodology, systematic reviews could be either quantitative (meta-analysis) or qualitative (1, 4). Meta-analysis is a quantitative statistical approach to aggregate the results of original studies. It can remarkably increase statistical strength, and precisely estimate the effect size, thus overcoming the limitation of sample size of individual studies (5–7). Additionally, it is able to explore the sources of heterogeneity as well as determine subgroups connected to the factor of interest (8). High-quality systematic reviews and meta-analyses (SRMAs) have potentials to underpin evidence-based clinical guidelines, and to inform decision-making. Therefore, well-conducted SRMAs are placed at the very top of the evidence pyramid in most current hierarchies (9). In the field of ophthalmology, reliable SRMAs have been increasingly identified and served as the backbone of the practice guidelines, such as the Preferred Practice Pattern (PPP) issued by the American Academy of Ophthalmology (10, 11). A prior survey in 2012 reported that SRMAs in ophthalmology had been actively performed, particularly in the domains of retina and glaucoma (12). Nonetheless, great advances have been taking place thereafter in evidence-based medicine, including the improvement of methodology for meta-analysis, adoption of principles of evidence-based medicine in precision medicine, and the implementation of prospective registration. For example, the Preferred Reporting Items for Systematic Reviews and Meta-Analyses (PRISMA) statements (13) were first released in 2009 and were updated lately (14). In 2011, the United Kingdom Centre for Reviews and Dissemination (CRD) at the University of York in England launched an international platform for prospective register of systematic reviews, PROSPERO (15), which aims to minimize reporting bias, reduce waste from unintended duplication, and increase transparency of SRMAs. Overall, these efforts have greatly helped standardize and improve the quality of SRMAs.

Bibliometric analysis is a quantitative method which applies mathematical and statistical tools to evaluate the inter-relationships and impacts of publications, authors, institutions and countries in a specific research area (16). Through extracting and analyzing the metrics of each publication including author, institution, country, and keywords, bibliometric analysis is able to determine the development trends or future research directions. Compared with conventional narrative reviews by experts, which often subjectively focus on the progress in a specific research field, bibliometric analysis is advantageous in objectively, comprehensively, and quantitatively summarizing the whole topic based on the information best available (16). More important, with various visualization approaches, the results are displayed in more intuitive and comprehensible ways, which enables scholars to gain a one-stop overview, identify knowledge gaps, uncover emerging trends, and explore the intellectual structure of a specific domain. Given these advantages, bibliometric analysis has gained immense popularity in biomedical research in recent years. For instance, there have been several bibliometric analyses in specialties of medicine and dentistry comprehensively depicting the research trends and hotspots (17–20). In the field of ophthalmology, a number of bibliometric analyses have been published regarding to certain ocular diseases (21, 22), and treatment or diagnostic modalities (23, 24). However, none has done on all available SRMAs in ophthalmology.

We herein performed this bibliometric analysis on SRMAs in ophthalmology published within the last two decades to explore the trends and patterns of publication, tracking impact and collaboration at author-, institution- and country-levels. These data would help ophthalmologists and scholars grasp the current state of development characteristics of the domain and guide future ophthalmological evidence-based research.

## 2. Methods

### 2.1. Data sources and search strategies

We designed a two-step approach to retrieve all relevant publications from 2000 to 2020. The first step aimed to extract all possible keywords in varying ophthalmological subspecialties. To this end, we searched the Web of Science-Core Collection (WoSCC) database which is maintained by Clarivate Analytics^1^ using the following parameters: TS = (“meta analysis” OR “meta analyses” OR “systematic review” OR “systematic reviews”), time span = “from 2000 to 2020,” language = “English,” Web of Science category = “Ophthalmology,” type = “article, review or early access.” TS here represents topic, meaning the search of the mentioned words in the title, abstract, and keyword lists. In this step, a total of 1,498 publications were obtained. We downloaded all original records and analyzed frequency of author’s keywords using the R package - *bibliometrix*. Keywords that had an occurrence ≥3 times were arbitrarily defined as the core ophthalmology-related keywords, yielding 128 terms (Supplementary material). Based on this collection, we conducted the second-round search in the WoSCC database. The retrieval strategy was set as: TS = (each of the 128 core keywords) AND TS = (“meta analysis” OR “meta analyses” OR “systematic review” OR “systematic reviews”). Other parameters, including publication year, language and literature type, were set identical to those in the first-round search. Both literature retrieval and raw data collection were performed on a single day (October 1, 2021).

### 2.2. Data cleaning

Raw metadata comprising 5,063 records in the 2nd round of literature search were downloaded from WoSCC. The dataset contained complete information of each publication for bibliometric analysis, including literature title, abstract, author list, journal name, keywords, publication year, countries/regions, affiliations, reference list, and citations. To assess eligibility of literature, the raw dataset was transformed and exported to Microsoft Excel 2017 using *bibliometrix* package, and then independently evaluated by two investigators (W.X. and Y.F.). Reports meeting any of the following criteria were excluded: (1) non-English publication; (2) publication year beyond 2000–2020; (3) retracted article. Full texts were then gone through to classify the subspecialty, and to evaluate whether they had pre-registered and/or adhered to reporting guidelines. Pre-registration was defined if the study prospectively registered its protocol on any of the major public platforms (25), including PROSPERO, the Registry of Systematic Reviews/Meta-Analyses in Research Registry, INPLASY, and Open Science Framework (OSF) Registries and protocols.io. Guideline compliance was assessed through checking whether the study used widely accepted statements, guidelines or checklists, like PRISMA and its extensions (PRISMA-IDP, PRISMA-NMA, etc.), and Meta-analyses Of Observational Studies in Epidemiology (MOOSE). Ophthalmological subspecialty was classified as 12 sections according to the subcategories listed by EyeWiki,^2^ an online resource launched by the American Academy of Ophthalmology Academy. Any disagreement between two raters was resolved through discussion. The cleaned dataset containing 2,660 publications were finally used for the subsequent bibliometric analysis.

### 2.3. Bibliometric analysis and visualization

Bibliometric analysis was conducted using the R-package *bibliometrix* and its shiny web-interface *biblioshiny* (26). Their integrative and powerful functions allow scholars easily conduct various scientometric analysis, from data importing and conversion, filtering, to various analytics and plots for different levels of metrics. The cleaned dataset was imported to *biblioshiny* to generate descriptive statistics, including the productivity and citation by author, affiliation, and country. Authors collaboration was illustrated with a network plot using the function biblioNetwork of *bibliometrix*. Only co-authorship ≥20 was retained in the network. Community structure within the network was uncovered using the Louvain clustering algorithm.

### 2.4. Statistical analysis

Descriptive statistics were directly extracted from the R-package *bibliometrix*. The proportions of pre-registered study and guideline complied study in ophthalmology and non-ophthalmology journals were compared with Pearson’s Chi-squared test. All statistical analyses and data visualization were performed using the R programming language (version 4.0.5) on RStudio’s open-source software (RStudio, Boston, MA). A *p* value <0.05 was considered as statistically significant.

### 2.5. Patient and public involvement

No patient was involved in this study.

## 3. Results

### 3.1. Characteristics of identified literature

**Figure 1:**
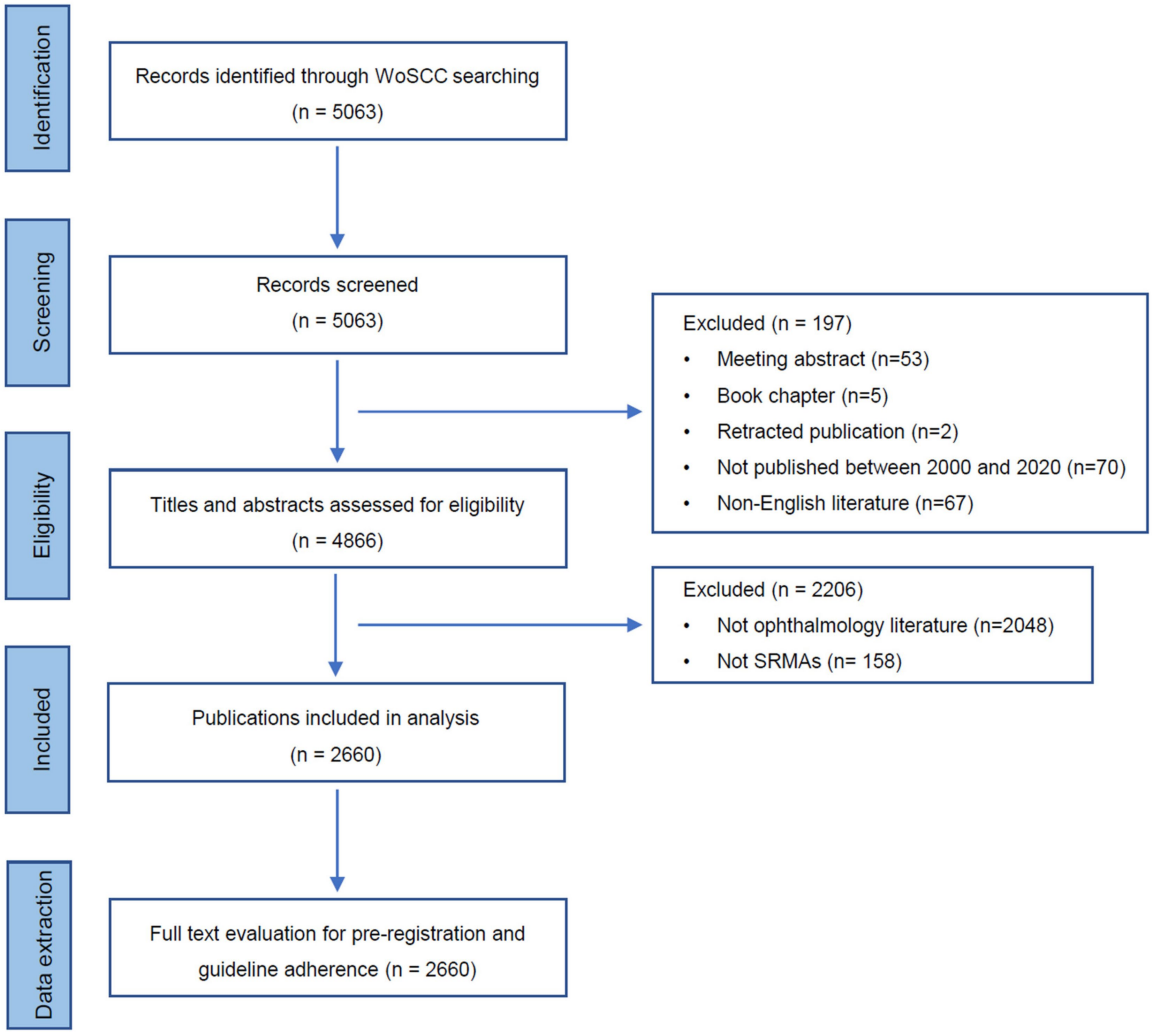
Flow diagram demonstrates the screening process of systematic reviews and meta-analyses in ophthalmology.

The process of literature identification was illustrated in Figure 1. A total of 5,063 records were initially obtained from the WoSCC database. After excluding ineligible items, 4,866 records were manually assessed for titles and abstracts. We ruled out non-ophthalmology literatures (*n* = 2,048) and non-SRMAs (*n* = 158), yielding 2,660 SRMAs for final analysis.

### 3.2. Productivity and impact by author and institution

**Figure 2:**
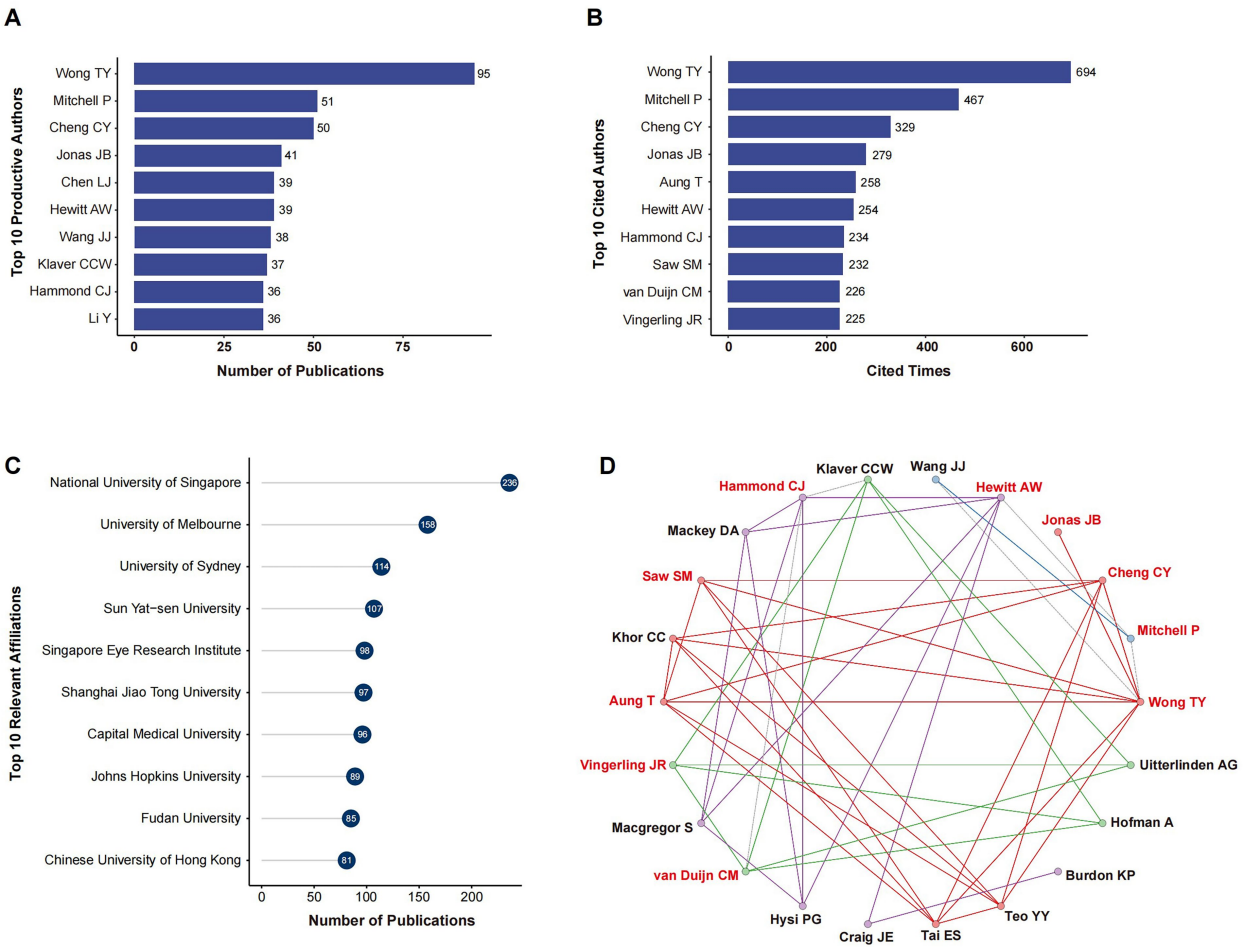
Author production and collaboration. **(A)** Top 10 productive authors. **(B)** Top 10 productive authors. **(C)** Top 10 relevant affiliations. **(D)** Collaboration network at author-level. The thickness of the line is proportional to the strength of co-authorship. Top 10 productive authors are highlighted in red.

The amount of literature had consistently increased over time, and the average annual growth rate was 21.26%. A total of 9,522 authors contributed to all 2,660 publications, resulting in average of 0.28 documents per author. The top 10 productive authors were: Wong TY (*n* = 95), Mitchell P (*n* = 51), Cheng CY (*n* = 50), Jonas JB (*n* = 41), Chen LJ (*n* = 39), Hewitt AW (*n* = 39), Wang JJ (*n* = 38), Klaver CCW (*n* = 37), Hammond CJ (*n* = 36), Li Y (*n* = 36) (Figure 2A). There was apparent overlap between top 10 cited and productive authors (Figure 2B). The most frequently cited author was Wong TY (*n* = 694), followed by Mitchell P (*n* = 467), Cheng CY (*n* = 329), Jonas JB (*n* = 279), Aung T (*n* = 258), Hewitt AW (*n* = 254), Hammond CJ (*n* = 234), Saw SM (*n* = 232), van Duijn CM (*n* = 226), Vingerling JR (*n* = 225). In terms of the top 10 relevant affiliations, National University of Singapore produced the most publications (*n* = 236); other highly productive affiliations included University of Melbourne (*n* = 158), and University of Sydney (*n* = 114, Figure 2C). The collaboration patterns at author-level were analyzed with *bibliometrix* package. The overall collaboration index was 3.63. Authors with extensive collaboration fell into an interconnected network (Figure 2D). Notably, all top 10 productive authors appeared in this network (Figure 2D, highlighted in red).

### 3.3. Country production

**Figure 3:**
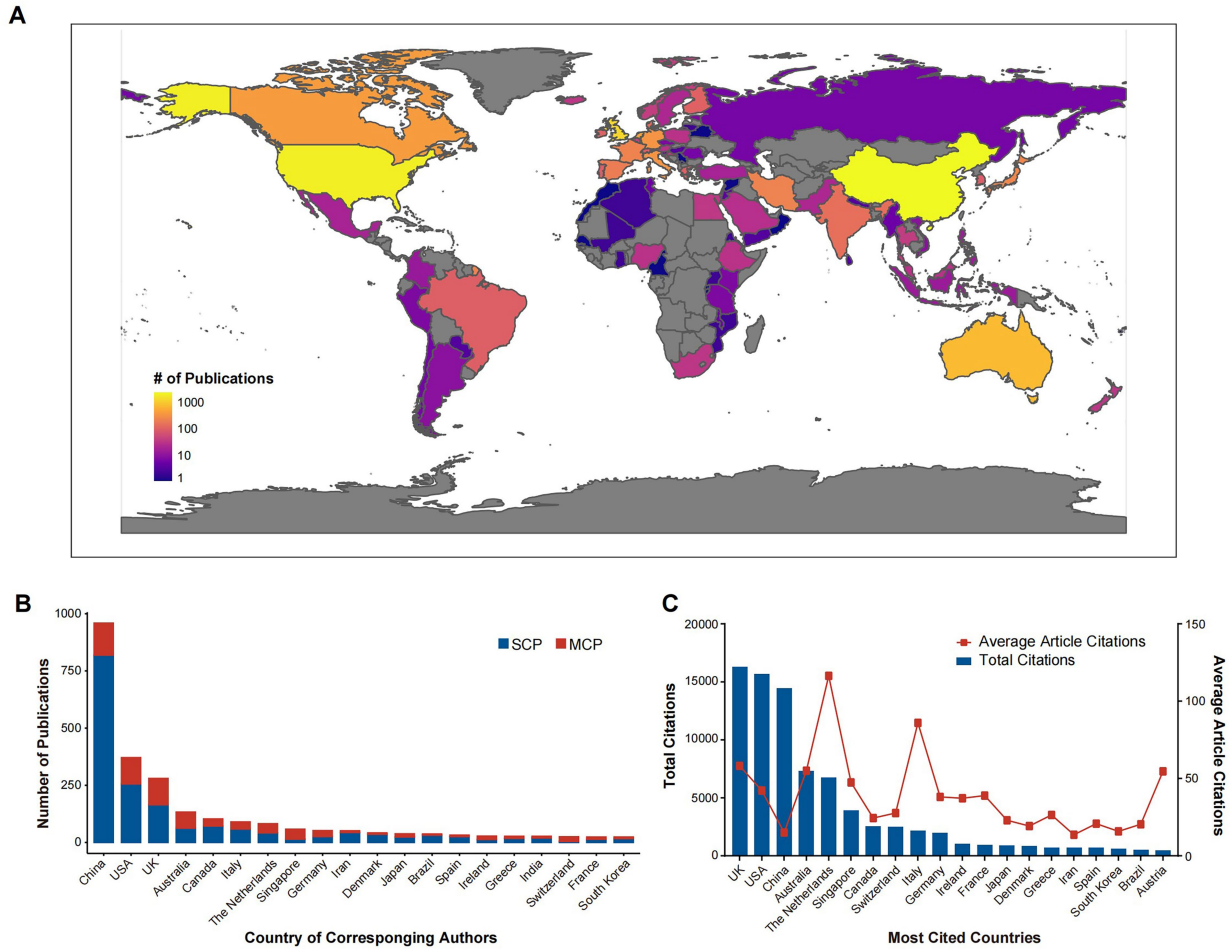
Country production and citation. **(A)** A heat map is presented displaying the number of publications from different countries according to the occurrence of authors. **(B)** Corresponding author’s country. **(C)** Top 10 cited countries. SCP, single country publication; MCP, multiple country publication.

There were 88 countries/regions that contributed to publish ophthalmological SRMAs from 2000 to 2020. A heat map visualized the geographical distribution of publication by country (Figure 3A). Overall, the United States and China were two countries with the most publications, other productive countries including those in the East Asia & Pacific, Europe & Central Asia, North America. However, countries in Latin America & Caribbean, Middle East & North Africa, South Asia and Sub-Saharan Africa were extremely underrepresented. As for corresponding authors’ country, we found that China ranked first with nearly 1,000 publications, followed by the United States, the United Kingdom, Australia and Canada (Figure 3B). These top 5 countries published approximately 70% of all articles. The percentage of multiple country publication (MCP) was used to reflect the inter-country collaboration. The vast majority were single country publication (SCP) in most top productive countries, including China, the United States and the United Kingdom. Of note, great percentages of MCP were observed in Singapore and Switzerland. Regarding the citation of SRMAs, we found that the United Kingdom, the United States and China were the top three most-cited countries with over 10,000 citations per country, followed by Australia and the Netherlands (both >5,000 citations) (Figure 3C). As for average citations per document, top five countries were Singapore (116.38 times), Switzerland (86.04 times), the United Kingdom (58.21 times), Australia (55.04 times), and Austria (54.62 times).

### 3.4. Ophthalmological subspecialties

**Figure 4:**
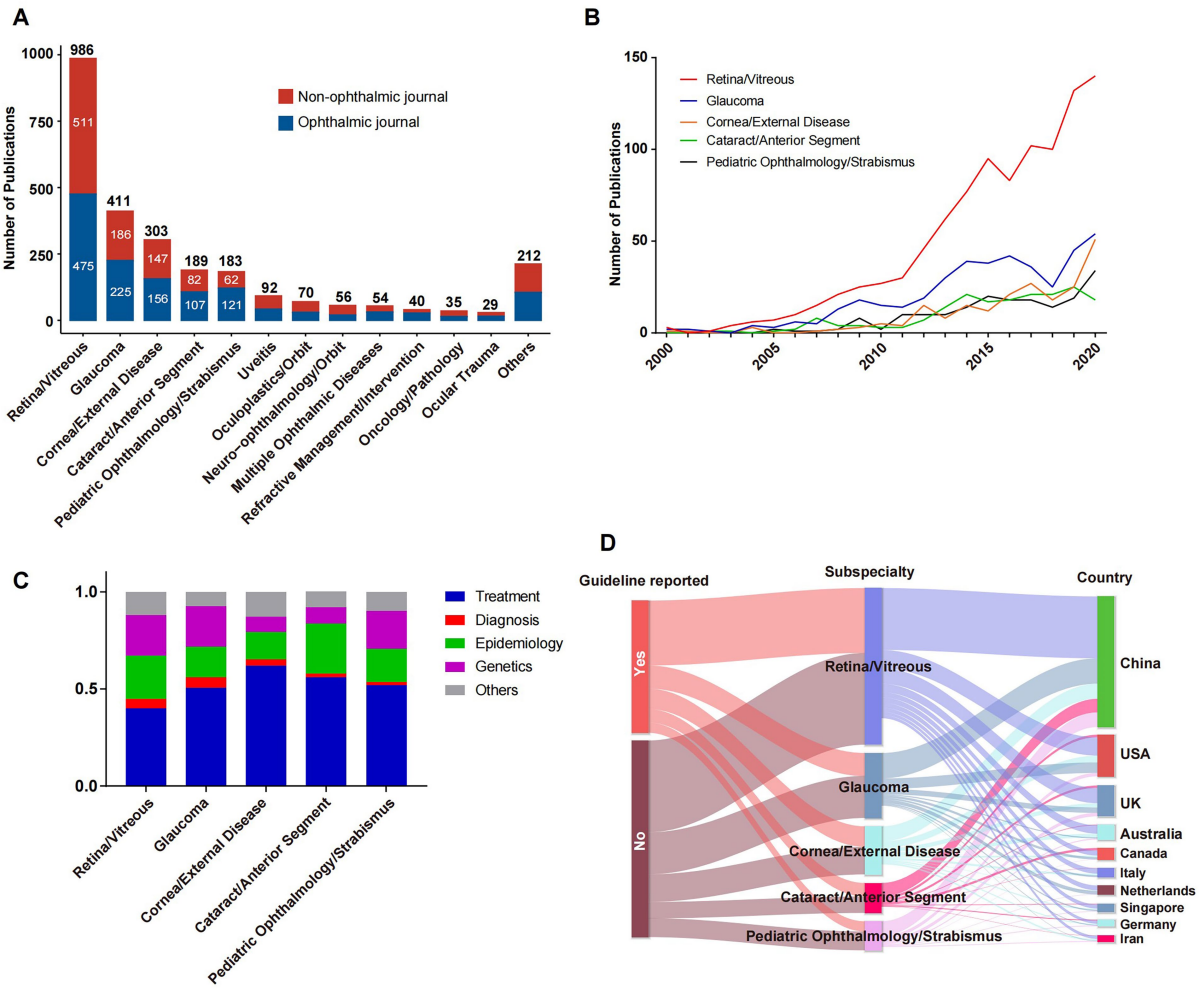
Publication in subspecialties. **(A)** Numbers of publications in different ophthalmological subspecialties. **(B)** The trends of publications in five major ophthalmological subspecialties from 2000 to 2020. **(C)** The proportion of different types of study content in five major ophthalmological subspecialties. **(D)** A Sankey diagram reveals the relationship between subspecialties and different bibliometric indicators.

The counts of SRMA in various ophthalmological subspecialties were listed in Figure 4A. Retina/vitreous (*n* = 986), glaucoma (*n* = 411), cornea/external diseases (*n* = 303), cataract/anterior segment (*n* = 189), and pediatric ophthalmology/strabismus (*n* = 183) were top five represented subspecialties. Approximately half of SRMAs were published in ophthalmology journals in most subspecialties, with an exception of Oncology/Pathology and Neuro−ophthalmology/Orbit, where SRMAs were more frequently published in non-ophthalmology journals. Year wise publication in various subspecialties was shown in Figure 4B. The number of publications in most subspecialties show a rising trend in general, especially after 2011 (Figure 4B). We then analyzed the proportion of different types of study content (Figure 4C) in major subspecialties. Treatment related SRMA accounted for about a half in each subspecialty, followed by those on epidemiology, genetics, and diagnosis. A Sankey plot revealed the relationship between subspecialties and various bibliometric properties (Figure 4D).

### 3.5. Pre-registration and guideline-compliance of systematic reviews and meta-analyses in ophthalmology

**Figure 5:**
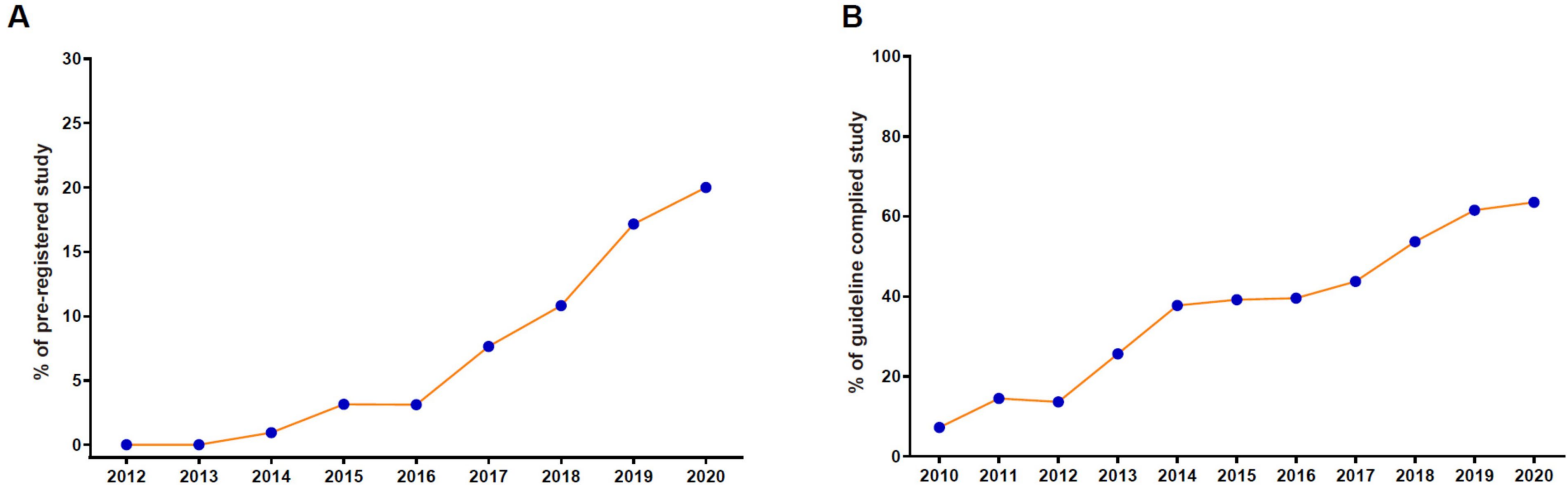
The percentage of pre-registered studies **(A)** and guideline complied studies **(B)** indexed by web of science core collection over time.

The percentage of pre-registered studies increased dramatically from 0 in 2012 to 20.0% in 2020 (Figure 5A). The rising trend significantly accelerated after 2016. Similarly, a gradual increase showed in the percent of guideline-complied studies from 2010 onwards, which reached up to 63.5% in 2020 (Figure 5B). There showed significantly lower percentages of pre-registered and guideline-complied studies published on ophthalmology journals compared with those on non-ophthalmology journals (Table 1, 5.6% vs. 10.2% for pre-registration, 36.6% vs. 45.1% for guideline-compliance, both *p* < 0.001).

**Table 1:** Percentage of pre-registered and guideline complied studies published on ophthalmology versus non-ophthalmology journals.

Characteristics	Journal category		*p*-value
	Ophthalmology (*n* = 1,368)	Non-ophthalmology (*n* = 1,292)	
Pre-registered (*n*, %)			
Yes	77 (5.6)	132 (10.2)	<0.001
No	1,291 (94.4)	1,160 (89.8)	
Guideline complied (*n*, %)			
Yes	501 (36.6)	583 (45.1)	<0.001
No	867 (63.4)	709 (54.9)	

## 4. Discussion

The present study aimed to construct the scientometric landscape of SRMA publications in ophthalmology over the period 2000 to 2020. Our data revealed the annual output gradually increased in ophthalmology as a whole, and also in most ophthalmological subspecialties, particularly in the domains of retinal/vitreous, glaucoma and cornea/external disease. China and the United States were the most productive countries, whereas Singapore was the country having the most prolific and influential scholar and institution. International collaboration was intense among high-impact authors. Finally, we found that the rates of pre-registration and reporting guideline compliance in ophthalmology SRMAs have been steadily increasing since 2012, yet leaving room for improvement.

The number of published SRMAs in ophthalmology has substantially increased over the past two decades. The average annual growth rate of publication was 21.26%, approximately 5-fold greater than the growth of overall scientific publication (4.10%) (27). We observed a striking 47-fold longitudinal increase of literature indexed in WoSCC from 2000 to 2020. The reasons for such increase are to be determined, but may in part attribute to the proliferation of SRMAs conducted by more researchers worldwide, especially those in China, as commonly seen in other medical specialties (28).

Geographically, around 70% of SRMAs in ophthalmology were published by scholars in top five productive countries (i.e., China, the United States, the United States, Australia, and Canada). Of note, China ranked first in output of articles, making China the most productive country on SRMA research in ophthalmology. In parallel, five out of top 10 productive affiliations were from China. Compared to rank 3^rd^ of China in 2010 (12), one can readily discern that China has experienced a rapid growth of production within the latest decade (2010–2020), exceeding any other countries. Similar findings were also observed in several other medical specialties than ophthalmology (29, 30). However, increased research output in China did not lead to simultaneous increase in international collaboration and the academic influence, as explicitly indicated by low percentage of MCP (15%) and low average citation per publication. In terms of the impact of country-level, a noteworthy country is Singapore. It has the most productive institution, numerous high-yield and high-impact scholars, and a close network of collaborations among these scholars, altogether making outstanding contributions to the application of evidence-based medicine in ophthalmology.

Not surprisingly, the field of retina/vitreous section remains the most intensely researched area in evidence-based medicine. Indeed, two out of top five leading causes of global blindness in people aged ≥50 years were retinal diseases (age-related macular degeneration, and diabetic retinopathy) (31). In the past two decades, clinical study on fundus diseases, especially on DR and ARMD, has been the focus of global ophthalmological research, with extensive investment of manpower, financial resources and funds. It is foreseeable that this trend will continue in the future.

The mass proliferation of SRMA publication has raised concerns about the quality and rigor of reports. A study estimated that only 3% of SRMAs are methodologically sound, non-redundant, thus provide useful clinical information (32). To minimize bias and increase transparency, systematic reviews are best to be prospectively registered on one hand, and are strictly adhered to set reporting guidelines on the other. Prospective registration on public platforms (e.g., PROSPERO) is a means to publish details about a research project before its commencement thus allowing evidence users to assess whether all steps of the research have been performed and reported as planned. Complete reporting, adhering to guidelines, for example PRISMA, allows readers to assess the appropriateness of the methods, and therefore the trustworthiness of the findings. In ophthalmology, our data showed that the percentages of pre-registered and guideline compliant SRMAs have significantly increased since 2012. This might be largely attributed to the fact that some scientific journals mandatorily require the authors to include a completed checklist in their submission to aid the editorial process and reader. As the rates of pre-registered and guideline compliant SRMAs were still much lower in ophthalmology journals than those in general medical journals, it is strongly recommended that ophthalmology journal editors to make the PRISMA checklist and pre-registration mandatory for all submissions of SRMAs to ensure their reliability and rigor. Further work to understand the barriers to pre-registration and guideline compliance uptake in ophthalmology is also required to address the gap identified by this study.

There has been an increasing number of bibliometric analyses on certain ocular disease or treatment modality of ophthalmology in recent years (33–39). These documents systematically revealed the productivity as well as collaborations of institutions, journals, and countries, making monitor the development of a specific field possible. Furthermore, they helped researchers or clinicians to master the research trends precisely and quickly, thereby aiding in conduct further studies. However, only a handful of bibliometric analyses focused on field of ophthalmology as a whole, and investigate the distribution and gap across subspecialties. In 2012, Chen et al. (12) analyzed the SRMAs published in ophthalmology from 1988 to 2010, and found the most heavily reported topics being retina and glaucoma. More than half of SRMAs were published in ophthalmology journals (about 60%). These trends have remained the same during the past decade. As this study shown, the most representative topics as well as the proportion of SRMAs published in ophthalmological journals almost unchanged. Another more recent bibliometric analysis investigated all available ophthalmological literature from 2017 to 2021, and found that epidemiology, prevention, screening, and treatment of ocular diseases were served as the hotspots. Moreover, artificial intelligence, drug development, and fundus diseases were acted as new research trends (40), indicating that non-ophthalmology knowledge were increasingly involved in ophthalmology research in the recent 5 years. In parallel, SRMAs on these themes are expected to increase in future.

Several limitations of this study should be addressed. First, one should note that some studies might have actually been compliant with guidelines regardless of no mention of using any specific statements in their full texts. Conversely, studies that stated use of particular guidelines may not necessarily satisfy all specific domains of its statement. As in-depth scoring and evaluating the quality of each SRMA using specific tools (e.g., A Measurement Tool to Assess systematic Reviews [AMSTAR] (41)) is beyond the scope of this study, future research is warranted to perform such analysis focusing on a particular subject. Second, we only searched WoSCC database which may not encompass the entirety of SRMA literatures in ophthalmology.

## 5. Conclusion

The annual output of SRMAs has been rapidly increasing over the past two decades. China and the United States were the most productive countries, whereas Singapore had the most prolific and influential scholar and institution. Raising awareness and implementation of SRMAs pre-registration and guideline compliance is still necessary to ensure quality, especially for ophthalmology journals.

## Data availability statement

We would like to remain the previous data availability statement, “Original data were obtained from the Web of Science-Core Collection (WoSCC) database. Dataset of the present study was available from the Dryad repository (http://datadryad.org/) with the link: https://doi.org/10.5061/dryad.fxpnvx0vw.

## Author contributions

WX and XC: conception, design, and administrative support. WX, YF, and SJ: data analysis and interpretation. YF, YM, SJ, SL, XC, and WX: manuscript writing, collection, and assembly of data. All authors read and approved the final manuscript.

## Funding

This study was supported by the National Natural Science Foundation of China (81600751); the Natural Science Foundation of Guangdong Province, China (2017A030313613, 2016A030310230); and the Pearl River Nova Program of Guangzhou (201806010167).

## Conflict of interest

The authors declare that the research was conducted in the absence of any commercial or financial relationships that could be construed as a potential conflict of interest.

## Publisher’s note

All claims expressed in this article are solely those of the authors and do not necessarily represent those of their affiliated organizations, or those of the publisher, the editors and the reviewers. Any product that may be evaluated in this article, or claim that may be made by its manufacturer, is not guaranteed or endorsed by the publisher.

## Footnotes

^1^https://www.webofknowledge.com

^2^https://eyewiki.org
