# Supplementary material for: A bibliometric analysis of systematic reviews and meta-analyses in ophthalmology
Source: Front Med (Lausanne). 2023 Mar 2;10:1135592. doi: 10.3389/fmed.2023.1135592 (PMC10017479; doi:10.3389/fmed.2023.1135592)
Supplement: Supplementary file 1 [file Data_Sheet_1.PDF]

Words	Occurrences
glaucoma	63
age-related macular degeneration	57
myopia	46
diabetic retinopathy	44
intraocular pressure	32
keratoconus	23
cataract	22
diabetic macular edema	20
retinal vein occlusion	20
vitrectomy	19
phacoemulsification	16
trabeculectomy	16
optical coherence tomography	15
dry eye	14
high myopia	14
retina	14
cataract surgery	13
macular hole	13
pterygium	13
visual acuity	13
amblyopia	12
macular degeneration	12
ocular hypertension	12
uveitis	12
cornea	11
macular edema	11
open angle glaucoma	11
photodynamic therapy	11
dry eye disease	10
blindness	9
internal limiting membrane	9
polypoidal choroidal vasculopathy	9
retinopathy of prematurity	9
visual impairment	9
age-related cataract	8
age-related macular degeneration (amd)	8
choroidal thickness	8
internal limiting membrane peeling	8
pars plana vitrectomy	8
primary open-angle glaucoma	8
refractive error	8
retinal detachment	8
axial length	7
central serous chorioretinopathy	7
choroidal neovascularization	7
neovascular age-related macular degeneration	7
ocular surface	7
orthokeratology	7
vision	7

geographic atrophy	6
intraocular lens	6
lasik	6
myopic maculopathy	6
open-angle glaucoma	6
pathologic myopia	6
primary open angle glaucoma	6
retinal nerve fiber layer	6
412 age-related macular degeneration	5
499 diabetic retinopathy	5
astigmatism	5
central corneal thickness	5
central retinal vein occlusion	5
dmek	5
endophthalmitis	5
endothelial keratoplasty	5
glaucoma drainage device	5
intravitreal injection	5
meibomian gland dysfunction	5
optical coherence tomography angiography	5
penetrating keratoplasty	5
poag	5
proliferative diabetic retinopathy	5
small incision lenticule extraction	5
strabismus	5
uveal melanoma	5
vision impairment	5
688 retina	4
conjunctiva	4
conjunctivitis	4
corneal collagen cross-linking	4
dry eye syndrome	4
dsaek	4
exfoliation syndrome	4
graves' ophthalmopathy	4
hyperopia	4
myopia progression	4
nasolacrimal duct obstruction	4
phacotrabeculectomy	4
primary angle-closure glaucoma	4
retinal nerve fiber layer thickness	4
retinoblastoma	4
rhegmatogenous retinal detachment	4
viscocanalostomy	4
vision loss	4
445 cataract	3
605 myopia	3
angle-closure glaucoma	3
best-corrected visual acuity	3
corneal sensitivity	3

corneal thickness	3
corneal transplantation	3
cyclophotocoagulation	3
deep sclerectomy	3
eye diseases	3
eye injuries	3
idiopathic macular hole	3
intermittent exotropia	3
intracorneal ring	3
intravitreal	3
intravitreal aflibercept	3
intravitreal ranibizumab	3
inverted internal limiting membrane flap technique	3
keratoconjunctivitis sicca	3
low vision	3
myopia control	3
myopic foveoschisis	3
neovascular glaucoma	3
nonpenetrating glaucoma surgery	3
optic neuritis	3
photorefractive keratectomy	3
posterior vitreous detachment	3
progressive keratoconus	3
retinitis pigmentosa	3
retinopathy	3
visual field	3
visual function	3
vitreomacular traction	3
vitreoretinal surgery	3
